# Practical implementation of an adaptive phase I/II design in chronic myeloid leukaemia: evaluating both efficacy and toxicity using the EffTox design

**DOI:** 10.1186/1745-6215-14-S1-P20

**Published:** 2013-11-29

**Authors:** Josephine Khan, Christina Yap, Richard Clark, Nicola Fenwick, David Marin

**Affiliations:** 1 Cancer Research UK Clinical Trials Unit, University of Birmingham, Birmingham, UK; 2 MRC Midland Hub for Trials Methodology Research, University of Birmingham, Birmingham, UK; 3 Department of Haematology, Royal Liverpool University Hospital, Prescot Street, Liverpool, UK; 4 Department of Haematology, Imperial College London, Hammersmith Hospital, London, UK

## 

Despite known limitations of algorithm based designs (e.g. 3+3) they are favoured for use in dose finding studies due to simplicity and familiarity. Bayesian adaptive designs can overcome these limitations as they’re more efficient and substantially more accurate.

EffTox is such a design which aims to determine the optimal, tolerable and efficacious dose. We implemented this design in a multicentre clinical trial of a novel TKI in combination with chemotherapy to treat chronic myeloid leukaemia. It is expected that the probability of efficacy may increase to a maximum then level off, thus higher doses of the combination may not result in greater efficacy. Given this non-monotone dose-response relationship, solely toxicity based dose-finding designs are not appropriate. In addition EffTox has demonstrated favourable properties of exposing fewer patients to potentially toxic and inefficacious doses, allocates more patients to the optimal dose and results in less dose-modifying toxicities in a subsequent phase-II/III trial.

A Bayesian analysis is conducted using a bivariate binary probability model with prior probabilities elicited from clinicians. Dose recommendations are based on an efficacy-toxicity trade-off contour. Operating characteristics were explored via simulations of various scenarios using readily available EffTox software. The design has satisfactory operational characteristics and is flexible to be adapted trial specific requirements.

Challenges in implementing this design included overcoming clinicians’ preconceptions, obtaining clinician consensus to enable development of the desirability trade-off contour and educating committees to secure funding. Overcoming these challenges allows design of robust dose-finding trials establishing an optimal, rather than sub-optimal dose.

